# Multi-Dimensional Mechanisms and Druggability Optimization Strategies of Active Ingredients from Traditional Chinese Medicine in the Treatment of Ulcerative Colitis

**DOI:** 10.3390/ph19070977

**Published:** 2026-06-24

**Authors:** Qiqi Fan, Xuxing Wang, Haixia Zhang, Zehua Chang, Na Wang, Shuo Fan, Zheng Li, Xinfang Xu, Chongjun Zhao, Xiangri Li

**Affiliations:** 1 School of Chinese Materia Medica, Beijing University of Chinese Medicine, Beijing 100029, China; 2 Beijing Key Laboratory for Quality Evaluation of Chinese Materia Medica, Beijing 100029, China; 3 Traditional Chinese Medicine Processing Technology Inheritance Base of National Administration of Traditional Chinese Medicine, Beijing University of Chinese Medicine, Beijing 100029, China

**Keywords:** TCM active ingredients, ulcerative colitis, molecular target, delivery strategies

## Abstract

Ulcerative colitis (UC) is a chronic inflammatory bowel disease characterized by a complex etiology and a protracted disease course. Active ingredients from traditional Chinese medicine (TCM), by leveraging the holistic regulatory advantages of anti-inflammatory activity, immune barrier preservation, and gut microbiota regulation, have shown unique therapeutic potential in the intervention of UC. Although bottlenecks such as unclear targets, fragmented mechanisms of action, and poor druggability constrain the clinical translation of TCM active ingredients, current research efforts are dedicated to overcoming these obstacles. This article reviews the latest research progress (2021–2026) on TCM active ingredients for UC treatment. It analyzes the anti-UC mechanisms from three core dimensions: chemical diversity and pharmacodynamic characteristics, validation of direct targets, and indirect regulation through the “gut microbiota–metabolite” axis. Moreover, it emphasizes recent breakthroughs in druggability optimization technologies, including carrier-based nano drug delivery systems (NDDS), carrier-free NDDS, co-delivery NDDS, and prodrug design strategy. Research demonstrates that TCM active ingredients achieve therapeutic effects by modulating inflammatory signaling networks, restoring intestinal immune homeostasis, repairing the mucosal barrier, and remodeling the gut microenvironment. Simultaneously, the application of novel delivery strategies effectively resolves issues such as poor solubility, low oral bioavailability, and insufficient colon targeting. Finally, this review suggests that future research on TCM active ingredients for UC therapy should concentrate on systematically clarifying multi-level mechanisms and designing clinically translatable smart drug delivery strategies, aiming to provide a theoretical basis and practical reference for promoting TCM modernization and innovative UC drug development.

## 1. Introduction

Ulcerative colitis (UC), a digestive-system disorder characterized by persistent inflammation of the intestinal mucosa, has a complex etiology and a long-lasting, recurrent course. Patients with UC suffer from weight loss, abdominal pain, diarrhea, and rectal bleeding, which severely degrade the quality of life. Long-term chronic inflammation substantially elevates the risk of colorectal cancer. UC is recognized by the World Health Organization as a modern intractable disease [1,2,3]. The pathogenesis of UC involves a multifaceted network of multifactorial interactions, including genetic susceptibility, immune dysregulation, gut microbiota imbalance, and mucosal barrier defects. At present, various pharmacological treatment and intervention strategies are available for clinical management, such as aminosalicylates, corticosteroids, immunosuppressants, and biologics. Nevertheless, these methods have limitations, including adverse reactions associated with long-term use, primary or secondary poor response in some patients, and a high relapse rate after treatment discontinuation [4,5,6,7]. Moreover, existing drug treatments cannot fully intervene in the complex pathophysiological processes of UC. Therefore, developing safer and more effective therapeutic strategies has become an urgent and crucial issue.

Traditional Chinese medicine (TCM), which is deeply rooted in the holistic concept and the principle of syndrome differentiation and treatment, is characterized by multi-target and multi-pathway regulation. This characteristic is well aligned with the complex pathogenesis of UC. A multitude of clinical and basic research studies have verified that TCM formulas and their active ingredients demonstrate unique therapeutic potential in reestablishing the homeostasis of the gut microenvironment, restoring the integrity of the intestinal barrier, and regulating immune responses [8,9,10,11]. Nevertheless, the active ingredients of TCM present inherent pharmacokinetic drawbacks, including poor water solubility, low oral bioavailability, rapid in vivo metabolism, and insufficient colon targeting [12]. Additionally, exploring the anti-UC mechanisms of TCM active ingredients has long been restricted to “component–pathway” correlation studies, lacking direct target validation and systematic regulatory network analysis. As a result, these limitations have emerged as a crucial bottleneck impeding clinical translation. Nano drug delivery systems (NDDS), which encapsulate therapeutic agents within nanoscale carriers, have significantly overcome many limitations of conventional drug delivery and are now widely applied across various biomedical fields [13,14]. The integration of NDDS with TCM amplifies the pharmacological advantages of TCM components, enhancing their bioavailability, targeting capability, and controlled release profiles. TCM-derived NDDS are primarily classified into two categories: carrier-based and carrier-free NDDS. The application of carrier-based NDDS such as polymeric nanoparticles, liposomes, micelles, and hydrogels has markedly improved the solubility and bioavailability of various TCM active ingredients, including curcumin, luteolin, and quercetin, thereby providing critical technological support for enhancing the quality and efficacy of TCM formulations [15,16,17,18,19]. The emergence of carrier-free NDDS, including extracellular vesicles and self-assembled nanoaggregates [20], has paved a green and efficient new route for the innovative drug discovery and development based on TCM. Despite the progress achieved in TCM-based NDDS, substantial challenges remain to be addressed for accelerating the clinical translation. Collectively, the introduction of NDDS has infused new vitality and possibilities into the sustainable development of TCM within the context of contemporary science and technology.

This review systematically summarizes the latest research progress on TCM active ingredients for UC treatment over the past five years. Initially, the chemical diversity and pharmacodynamic characteristics of TCM active ingredients, including flavonoids, alkaloids, phenols, terpenoids, and polysaccharides, are outlined. Subsequently, an in-depth analysis of the research progress is summarized at two distinct levels: direct target discovery and validation, and indirect regulation through the gut microbiota–metabolite axis. Thirdly, it focuses on the recent progress in druggability optimization technologies, such as carrier-based nano drug delivery system (NDDS), carrier-free NDDS, co-delivery NDDS, and prodrug design strategy. Finally, the current challenges and future directions are discussed, providing new perspectives for promoting the modern development and clinical application of TCM active ingredients in UC therapy.

## 2. Multi-Dimensional Mechanisms of TCM Active Ingredients

### 2.1. Chemical Diversity and Efficacy Profiles of Active TCM Active Ingredients

**Figure 1:**
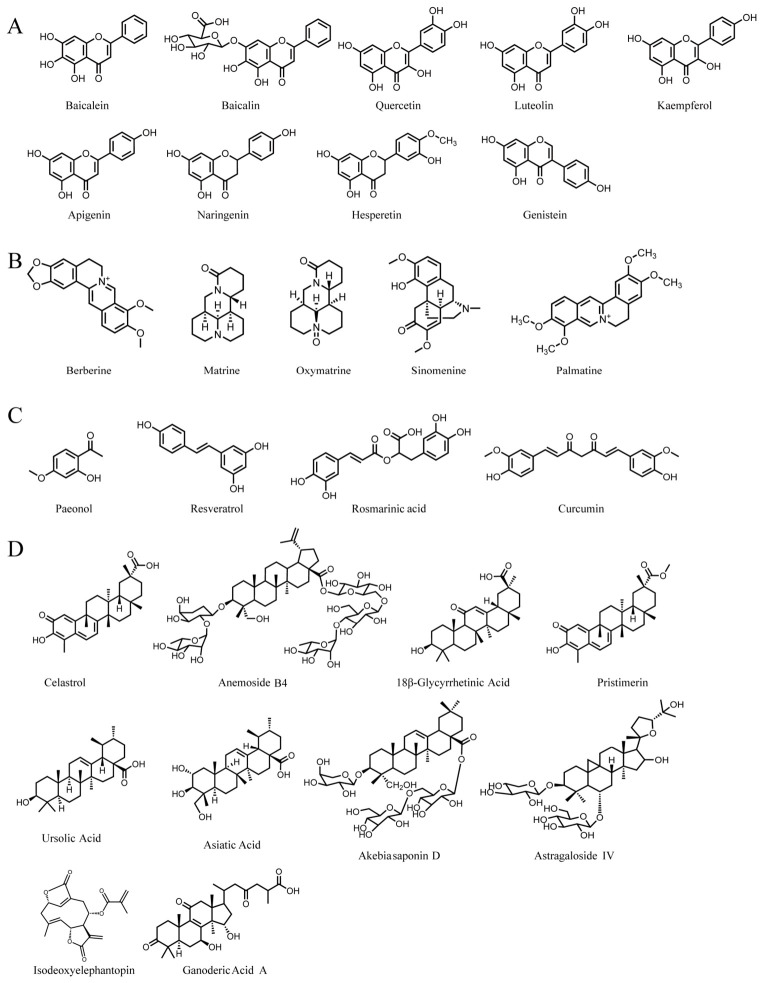
Structures of TCM active ingredients. (**A**) Flavonoids, (**B**) Alkaloids, (**C**) Phenols, (**D**) Terpenoids.

Structurally diverse active ingredients of TCM (Figure 1) have recently been investigated in anti-UC research, with a primary emphasis on five major classes: flavonoids, alkaloids, phenols, terpenoids, and polysaccharides. Currently, pharmacodynamic evaluations in vivo mainly utilize dextran sulfate sodium (DSS)-induced C57BL/6 or BALB/c mouse models. DSS strains, concentrations, and sources are key factors that can affect experimental results. Pharmacodynamic evaluation primarily encompasses the disease activity index (DAI), colon length, colonic histopathological changes, levels of inflammatory cytokines, and the expression of intestinal barrier-related proteins. Although various classes of TCM active ingredients exert anti-UC effects through multi-target and multi-pathway mechanisms, their respective pharmacodynamic characteristics and mechanistic focus differ to some extent, endowing each class with distinctive therapeutic advantages.

Flavonoids, as one of the most comprehensively studied active ingredients in TCM, possess a core therapeutic advantage: the dual effects of activating the aryl hydrocarbon receptor (AhR) to enhance intestinal barrier function and inhibiting the inflammatory signaling pathway such as nuclear factor kappa B (NF-κB), NLR family pyrin domain containing 3 (NLRP3), Janus kinase/Signal transducer and activator of transcription (JAK/STAT) to alleviate inflammatory responses. As a crucial hub connecting environmental signals, immune cells, and the intestinal epithelial barrier, AhR represents a potential therapeutic target for UC [21,22]. For example, quercetin activated AhR in intestinal epithelial cells to upregulate tight junction proteins (such as ZO-1 and Occludin) and repair the intestinal mucosal barrier, and baicalein promoted Interleukin-22 (IL-22) secretion via AhR in type 3 innate lymphoid cells (ILC3s), enhancing intestinal epithelial barrier function [23,24]. The NF-κB signaling system is composed of homodimers or heterodimers of NF-κB subunits, including RelA (p65), c-Rel, RelB, p50, and p52, as well as the inhibitory proteins IκBα, IκBβ, IκBε, p105, and p100. Pro-inflammatory signals (e.g., cytokine receptors of the IL-1R and TNF-R families) or Toll-like receptors activate the IκB kinase complex, which subsequently phosphorylates and ubiquitinates IκB proteins, targeting them for degradation, thereby ultimately activating NF-κB [25,26,27]. Flavonoids generally inhibit the activation of the NF-κB signaling pathway and reduce the release of pro-inflammatory cytokines such as TNF-α, IL-1β, and IL-6, thereby exerting broad-spectrum anti-inflammatory effects [28,29,30,31,32]. Activation of inflammatory signals, excessive release of reactive oxygen species (ROS), and Ca^2+^ overload can upregulate NLRP3. Flavonoids such as luteolin and kaempferol ameliorate intestinal inflammation and repair intestinal barrier damage by inhibiting NLRP3 [33,34,35]. Stimulation by inflammatory cytokines such as IL-6, IL-22, and IL-23 triggers downstream signaling pathways, leading to phosphorylation and activation of STAT3 in T cells and macrophages, thereby affecting immune regulation. Flavonoids such as baicalin, luteolin, and genistein ameliorate inflammation in UC by inhibiting STAT3, as reflected by the modulation of the Th17–macrophage axis, reduction in inflammatory cytokine secretion, and promotion of intestinal barrier restoration [25,36].

Alkaloids have played a crucial role in the regulation of immune cells, particularly in the modulation of the T helper 17 cell/Regulatory T cell (Th17/Treg) balance. As the most comprehensively studied isoquinoline alkaloid, berberine has demonstrated multi-level mechanisms by modulating the gut microbiota and restoring both the ILC1/ILC3 and the Treg/Th17 balance. This effect is reliant on the activation of the Wnt/β-catenin signaling pathway [37]. Matrine has corrected the Th17/Treg imbalance and regulated the gut microbiota by increasing the abundance of beneficial bacteria, such as *Lactobacillus* and *Akkermansia*, while inhibiting reducing the proportion of Proteobacteria [38]. Both alkaloids have indirectly regulated T-cell differentiation via the microbiota–immune axis, thereby suppressing intestinal inflammation and repairing the intestinal mucosal barrier.

Moreover, the anti-inflammatory effects of phenols were mainly achieved through the regulation of the NF-κB and PI3K/AKT signaling pathways [39,40,41,42]. Phosphatidylinositol 3-Kinase/Protein Kinase B (PI3K/AKT) is closely associated with the onset and progression of UC. Upon activation, PI3K catalyzes the conversion of phosphatidylinositol bisphosphate to phosphatidylinositol triphosphate, which subsequently activates the downstream effector molecule AKT, thereby influencing the secretion of inflammatory cytokines and participating in the regulation of NF-κB [25]. For instance, paeonol could upregulate the expression of Peroxisome proliferator-activated receptor gamma (PPARγ), inhibit the activation of NF-κB, decrease the production of pro-inflammatory cytokines, and concurrently reduce the levels of ROS [40]. Rosmarinic acid and resveratrol exert therapeutic effects against UC by modulating the PI3K signaling pathway [41,42].

Mechanistic investigations on terpenoids for UC treatment display diverse characteristics. These involve not only the regulation of inflammation-related signaling pathways, such as the NF-κB, MEK/ERK, and Wnt/β-catenin pathways, but also the reestablishment of immune homeostasis, along with the modulation of gut microbiota composition and associated metabolites. For instance, ganoderic acid A exerted anti-UC effects by inhibiting Th17 differentiation and M1 macrophage polarization to regulate immune responses. It also modified the gut microbiota composition, elevated 3-IAld levels, and activated AhR [43,44].

The anti-UC effects of polysaccharides are characterized by a strong reliance on gut microbiota modulation. Polysaccharides facilitate the proliferation of beneficial bacteria and enhance the production of short-chain fatty acids (SCFAs), thereby modulating the intestinal microenvironment. Moreover, it has been observed that polysaccharides demonstrate remarkable advantages in regulating immune cells. For instance, Astragalus polysaccharide restored the Th17/Treg cell balance, while *Lycium barbarum* polysaccharide and Dendrobium polysaccharides preferentially regulated the M1/M2 macrophage polarization balance [45,46,47]. Recent research has indicated that polysaccharides exert a comprehensive regulatory effect via the “microbiota–metabolism–immunity” axis, and the structure–activity relationship governing the interaction with specific gut microbiota stands as a crucial focus for future investigations.

**Figure 2:**
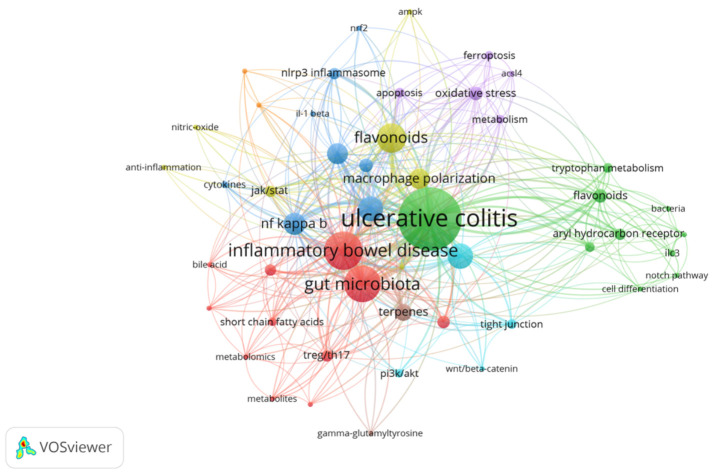
Bibliometric analysis of keyword co-occurrence in research on five major classes of TCM active ingredients for UC therapy (labels in different colors represent keywords). The network visualization was created in VOSviewer (v1.6.20).

**Figure 3:**
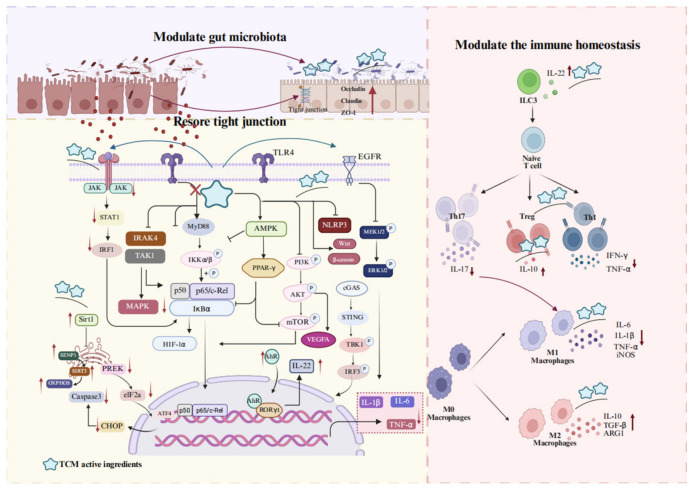
Summary of the main regulatory mechanisms of TCM active ingredients in UC treatment (↑ indicates upregulation or promotion, and ↓ indicates downregulation or inhibition). The figure was created in BioRender. Fan, Q. (2026) (https://BioRender.com/fdbf5qj, accessed on 27 May 2026).

Based on a bibliometric keyword co-occurrence analysis, a further integration of the research reports summarized in Table 1 indicates that although the anti-UC mechanisms of various components have different focuses, they ultimately converge into four core regulatory networks (Figure 2 and Figure 3). Overall, the active ingredients of TCM exhibit multi-dimensional regulatory mechanisms, including the modulation of inflammation, the regulation of immunity, remodeling of the gut microbiota and intestinal metabolites. Specifically, at the inflammatory level, the majority of TCM active ingredients reduce the release of pro-inflammatory cytokines (e.g., TNF-α, IL-1β, and IL-6) by regulating key pathways such as NF-κB, PI3K-AKT, NLRP3, and JAK/STAT. Furthermore, these signaling pathways are interlinked and mutually influence one another. On the immunological aspect, TCM active ingredients can reestablish intestinal immune homeostasis by restoring the Th17/Treg balance and the M1/M2 macrophage polarization balance, as well as regulating ILC3 cells. With respect to barrier integrity, these compounds strengthen the epithelial barrier function by upregulating tight junction proteins, including ZO-1, Occludin, and Claudin. Moreover, a growing body of evidence suggests that TCM active ingredients alleviate colonic inflammation and restore barrier homeostasis by modulating the abundance, metabolism (SCFAs, bile acids and so on), and diversity of gut microbiota. Of note, the intricate interrelationships among these regulatory mechanisms warrant further elucidation. Although the findings to date are promising, most of the current evidence remains confined to preclinical models, and its validation in large-scale, randomized controlled clinical trials is limited.

### 2.2. Deepening Mechanistic Exploration: Research on the Direct Targets of TCM Active Ingredients

**Figure 4:**
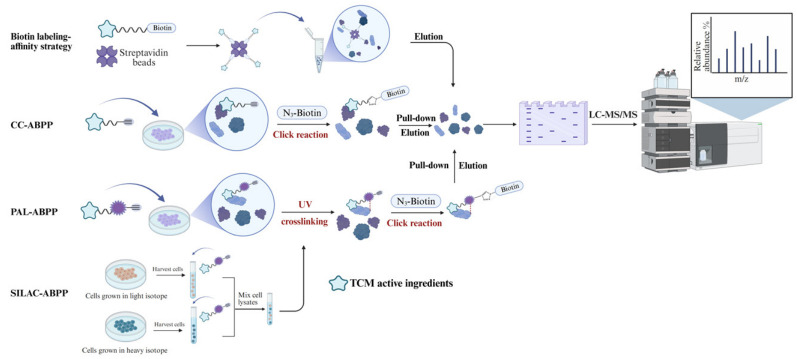
Labeled target discovery technology applied to TCM ingredients. The figure was created in BioRender. Fan, Q. (2026) (https://BioRender.com/kjut9xw, accessed on 27 May 2026).

**Figure 5:**
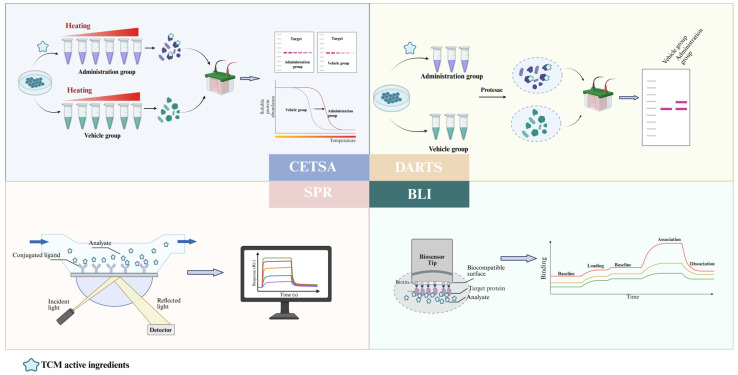
Label-free target discovery technology applied to TCM ingredients. The figure was created in BioRender. Fan, Q. (2026) (https://BioRender.com/2dw3u9i, accessed on 27 May 2026).

Over an extended period, mechanistic investigations into the active ingredients of TCM have predominantly remained at the stage of “component–pathway” correlation analysis. In recent years, with the rapid advancement of chemical biology, structural biology, and omics technologies, the technical framework for target discovery and validation has been gradually refined. This refinement has propelled anti-UC research in TCM from “pathway association” towards causal validation of “molecule-target” interactions, thus facilitating the elucidation of precise mechanisms. At present, the technologies employed for target discovery of TCM active ingredients are primarily classified into labeling-based and label-free methods (Figure 4 and Figure 5), which are frequently used in combination to improve the accuracy of target identification.

#### 2.2.1. Labeled Target Discovery Strategy for Target Identification

In the labeled target discovery strategy, biotin labeling represents the most commonly employed method for affinity purification in target identification. This approach depends on specific physical interactions between the ligand and the target, allowing for the capture of target proteins from cell or tissue lysates onto solid supports conjugated with avidin, followed by purification through gel electrophoresis and subsequent mass spectrometry analysis [103,104]. Activity-based Protein Profiling (ABPP), which has emerged in recent years, is a widely utilized target enrichment strategy in chemical proteomics. Its core principle entails designing activity-based small-molecule probes that capture target proteins via covalent binding, followed by gel electrophoresis or proteomics for further identification [105,106,107]. In situations where small molecules interact with target proteins through non-covalent binding, photoaffinity labeling (PAL) and click chemistry (CC) methods are introduced. PAL-ABPP employs photo-crosslinking groups (such as azidomethyl benzene, diazirine, and benzophenone), which generate highly reactive intermediates (e.g., nitrene, carbene, or diradical) upon ultraviolet irradiation, subsequently forming covalent bonds with proteins [108,109,110]. CC-ABPP, conversely, introduces bioorthogonal handles such as terminal alkynes or azide groups into the probes and then utilizes CuAAC or other CC reactions to conjugate the reporter tag, enabling specific labeling and enrichment of target proteins [109,111]. Moreover, stable isotope labeling by amino acids (SILAC)-ABPP uses stable isotope labeling in living cells and affinity tags to enrich target proteins for mass spectrometry quantitation [106], yet it is only applicable to in vitro cells, not tissue or clinical samples.

#### 2.2.2. Label-Free Target Discovery Strategy for Target Identification

Label-free target discovery strategy facilitates target identification without chemical modification of bioactive small molecules by capitalizing on the enhanced stability of the complex, which encompasses thermal stability, protease resistance, and altered physicochemical properties. Cellular Thermal Shift Assay (CETSA), initially proposed in 2013, presents a method for monitoring drug–target interactions at the cellular or tissue level [112]. This method is based on the principle that drug binding modifies the thermal stability of target proteins. In this approach, cells or lysates are incubated with the drug, followed by the application of a temperature gradient. Proteins bound to the drug remain soluble because of the increased thermal stability, whereas unbound proteins precipitate. Subsequently, the soluble fraction is analyzed via Western blot and LC-MS/MS to identify proteins with differential stability [113,114]. Drug Affinity Responsive Target Stability (DARTS) is a label-free target discovery technology. It is founded on the principle that ligand binding stabilizes target proteins, making them resistant to protease digestion, such as by thermolysin or pronase. The protected target protein is identified through a comparison of SDS-PAGE profiles [115,116]. Surface Plasmon Resonance (SPR) technology detects alterations in the refractive index at a metal-dielectric interface caused by molecular binding. This enables label-free, real-time analysis of molecular interactions and has established SPR as the “gold standard” for studying biomolecular interactions [117,118]. Bio-layer interferometry (BLI) is a label-free, real-time technique that monitors changes in optical thickness to measure binding kinetics (including association/dissociation rates and affinity constants). It is widely used to validate direct interactions between TCM active ingredients and target proteins [119,120,121,122]. In the studies of target identification for TCM active ingredients, label-free target strategies are often used in combination to eliminate non-specific interference (Figure 5), and the functionality of the targets is further validated through gene knockdown/knockout.

#### 2.2.3. Labeled and Label-Free Target Identifications in UC Treatment of TCM Ingredients

In recent years, the integrated application of the aforementioned technologies has effectively identified multiple direct targets of TCM active ingredients in the treatment of UC, offering direct evidence for clarifying the precise mechanisms. For example, Meng, G. et al. utilized SILAC-ABPP in combination with a photoaffinity probe to screen and identify immunity-related GTPase family M protein 1 (IRGM1) as the direct anti-inflammatory target, which was validated through CETSA, DARTS, BLI, and gene knockdown experiments. The results revealed that berberine exerted anti-inflammatory effects by targeting IRGM1 and regulating the PI3K/AKT/mammalian target of rapamycin (mTOR) pathway [68]. By using a biotin-labeled probe for protein pull-down, followed by mass spectrometry identification and validation with DARTS, CETSA, and SPR, Liang, Q.H. et al. demonstrated for the first time that Anemoside B4 directly bound to the key binding site His879 of pyruvate carboxylase (PC), which subsequently reprogrammed pyruvate metabolism and inhibited NF-κB signaling [87]. Moreover, apigenin has been recognized as a natural inhibitor of interleukin-1 receptor-associated kinase 4 (IRAK4) [61]; gingerenone A has been shown to directly bind to IL-17RA [123]; and akebia saponin D directly targeted epidermal growth factor receptor (EGFR) [92]. These findings not only elucidate the targets of TCM active ingredients but also present new candidate targets for precision therapy of UC.

The advancement of target validation technologies has driven a transformation in mechanistic research on TCM against UC, facilitating the clarification of the action mechanisms at the molecular level. Nevertheless, the direct targets of the majority of TCM active ingredients remain ambiguous, and current studies are deficient in in situ assessment of target binding within the UC pathological microenvironment, which represents a crucial direction for future mechanistic investigations.

### 2.3. A Shift in Research Perspective: Gut Microbiota-Dependent Regulatory Mechanisms of TCM Active Ingredients

**Figure 6:**
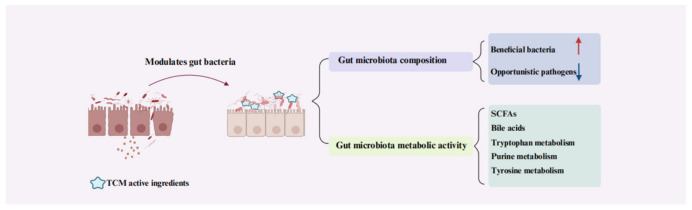
The regulation of gut microbiota and metabolic activities by TCM active ingredients (↑ indicates promotion, and ↓ indicates inhibition). The figure was created in BioRender. Fan, Q. (2026) (https://BioRender.com/cpvwf7x, accessed on 27 May 2026).

TCM active ingredients not only act directly on host cellular targets and pathways but also exert indirect therapeutic effects by modulating the gut microbiota and its metabolic activities (Figure 6). With the extensive application of metagenomics, metabolomics, and fecal microbiota transplantation (FMT), the “microbiota–metabolism–host” axis is now acknowledged as central to UC pathogenesis and a key focus in TCM research. This shift in research perspective deviates from the traditional host-centered model and offers a novel microecological intervention strategy for the treatment of UC.

#### 2.3.1. Reshaping Gut Microbiota Composition

The gut microbiota establishes a mutualistic symbiotic relationship with the host, which helps maintain homeostasis, regulate carbohydrate and vitamin metabolism, and support the development of the immune system [124,125,126]. The onset and progression of UC are closely linked to gut microbiota dysbiosis. Patients with UC display significant structural disruptions in the gut microbiota, characterized by a reduction in diversity and richness. At the phylum level, the abundance of Firmicutes declines, while that of Proteobacteria rises. Beneficial bacteria such as *Bifidobacterium* and *Lactobacillus* decrease, whereas opportunistic pathogens like *Escherichia coli* proliferate [127,128,129]. From Table 1, TCM active ingredients generally modulated the microbiota by reducing the abundance of Proteobacteria, promoting the growth of SCFAs-producing bacteria and probiotics (such as *Lachnospiraceae*, *Butyricicoccus*, *Rikenella*, *Akkermansia*, and *Ruminococcaceae*) and suppressing opportunistic pathogens (such as *Escherichia-Shigella* and *Bacteroides*). The aforementioned core intestinal commensal bacteria play critical roles in host immunity, regulation of inflammatory responses, and intestinal barrier function, primarily manifested in promoting SCFAs metabolism, particularly butyrate, modulating immune cell differentiation, and maintaining the intestinal mucosal barrier. For instance, *Akkermansia* can directly activate immune responses, influence the proportion of Treg cells in mesenteric lymph nodes (MLNs) and the spleen, and regulate γδT17 cell and macrophage polarization through the TLR2 signaling pathway [130,131]. Butyrate produced by commensal bacteria such as Ruminococcaceae and Lachnospiraceae regulates colonic Treg cells and macrophages, reduces the release of inflammatory cytokines, and promotes the restoration of epithelial barrier function by modulating HIF-1, STAT3, and SP1 [132,133]. Furthermore, specific components have differential effects on particular taxa. For example, luteolin enriched *Prevotellaceae* and *Clostridium_cocleatum*, matrine increased *Barnesiella intestinihominis* while inhibiting *Helicobacter gammali*, and pristimerin upregulated *Lachnospiraceae NK4A136_group* and *Alloprevotella*. This specific regulation implies selective interactions between the structures of TCM active ingredients and the gut microbiota, which warrants further investigation of the structure–activity relationship.

#### 2.3.2. Regulation of Metabolic Activities

The gut microbiota functions as a pivotal executor of host metabolism, with its metabolites acting as crucial bridges linking the microbial community to the host [134,135]. TCM active ingredients have the potential to reshape the composition of the gut microbiota and the intestinal metabolic profile, thereby regulating metabolic pathways and indirectly mitigating inflammation associated with UC. Contemporary research predominantly centers on the regulation of metabolic pathways related to SCFAs, bile acids, tryptophan, and tyrosine.

SCFAs, including acetate, propionate, and butyrate, are the primary metabolites generated by gut microbiota via the fermentation of dietary fiber. They are crucial for the energy supply of intestinal epithelial cells, the enhancement of mucosal defense mechanisms, and immune regulation [136]. TCM active ingredients can augment the abundance of SCFA-producing microbiota. For instance, ursolic acid has been shown to increase the abundance of *Clostridium_sensu_stricto_1* and *Dubosiella*, elevate propionic acid production, and consequently restore the Treg/Th17 immune balance [90]. Polysaccharides present unique advantages in reshaping SCFA metabolism. Astragalus polysaccharide [45], *Lycium barbarum* polysaccharide [98] and Dendrobium polysaccharide [99] could increase the production of SCFAs such as acetic acid, butyric acid, propionic acid, and isobutyric, thereby activating FFAR2/FFAR3 and PPARγ to exert anti-inflammatory effects.

Bile acids are significant metabolites synthesized in the liver and subsequently metabolized by the gut microbiota. They participate in the regulation of intestinal immune homeostasis by activating nuclear receptors such as FXR and TGR5 [137,138,139]. Berberine facilitated the increase in secondary bile acids (e.g., ursodeoxycholic acid, UDCA; deoxycholic acid, DCA) in the intestine and restored bile acid metabolism balance, activated the expression of FXR and TGR5, suppressed ERK phosphorylation in colon tissue, and repaired the intestinal mucosal barrier [71]. Abelmoschus polysaccharide enriched gut bacteria such as *Bacteroides* and *Blautia*, and increased metabolites including 7-ketodeoxycholic acid, thereby indirectly activating the FXR/STAT3 pathway [101].

The “microbiota–tryptophan metabolism–AhR” axis has garnered significant attention in the research regarding the anti-UC effects of active ingredients in TCM. Indole derivatives generated by the gut microbiota from tryptophan serve as endogenous ligands of the AhR and play a crucial role in intestinal barrier repair [140]. Through reshaping the tryptophan metabolic profile and increasing the levels of tryptophan derivatives such as indolelactic acid, quercetin indirectly activated the AhR signaling pathway, resulting in the repair of the intestinal mucosal barrier and the alleviation of UC [54]. By augmenting the abundance of *Lactobacillus* and influencing the tryptophan metabolism in the colon, ginseng polysaccharide inhibited the 5-HT/HTR3A signaling pathway, thereby enhancing intestinal barrier function and ameliorating colonic inflammation and injury [102].

Furthermore, the gut microbiota plays a crucial role in the metabolism of purines and uric acid, and an imbalance in uric acid metabolism not only induces well-known kidney diseases but also affects intestinal function [141]. Emodin has the potential to reduce uric acid levels through the modulation of purine metabolism, thus reversing the intestinal barrier damage induced by uric acid [142]. Palmatine exerted a bidirectional regulatory effect on gut microbiota and tyrosine metabolism. It inhibited *Bacteroides acidifaciens*, leading to a reduction in p-cresol (PC) and blocking PC-mediated Th17 differentiation and pro-inflammation. Meanwhile, it promoted *Bacteroides stercoriosoris*, resulting in an increase in p-hydroxyphenylacetic acid, thus remodeling the metabolic balance and synergistically alleviating colonic inflammation [143].

In summary, TCM active ingredients exert comprehensive anti-UC regulation by reshaping the gut microbiota structure and coordinately modulating multiple metabolite profiles, including SCFAs, bile acids, tryptophan, purines, and tyrosine. The shift in the research perspective from a “host-centered” to a “microbiota–host interaction” paradigm not only deepens the understanding of the mechanisms of TCM but also offers new insights for UC therapy based on microecological intervention. Despite these advances, most studies are limited to correlational evidence, and the complex interactions among diverse metabolites warrant further dissection and validation.

## 3. Carrier-Based NDDS for TCM Active Ingredients

Although the active ingredients of TCM demonstrate significant potential in the treatment of UC, the clinical translation is severely restricted by pharmacokinetic deficiencies, including poor water solubility, low oral bioavailability, rapid in vivo metabolism, and inadequate colon targeting [144]. Recently, diverse colon-targeted NDDS have been developed based on the microenvironmental characteristics of UC (e.g., pH, ROS, enzymes, and receptor overexpression), facilitating precise drug accumulation and controlled release at inflamed sites. Consequently, this approach enhances the efficacy and reduces the side effects of TCM active ingredients [145].

### 3.1. ROS-Sensitive NDDS

Persistent inflammatory stimulation results in ROS levels in the intestinal mucosa of UC patients that are 10–100 times higher than those in healthy individuals [146,147]. The elevated ROS levels in the mucosa offer an ideal trigger for ROS-responsive NDDS, which utilize carrier materials functionalized with ROS-labile linkages (e.g., thioether or diselenide bonds). These linkages cleave under high ROS conditions to achieve site-specific drug release. For example, an amphiphilic ROS-responsive copolymer synthesized by combining a new thioether containing poly(β-thioester) (PBTE) with D-α-tocopheryl polyethylene glycol succinate (TPGS) was employed to encapsulate luteolin, leading to the formation of LUT@TPGS-PBTE nanoparticles. In a high-ROS microenvironment, the oxidation of thioether bonds expedited drug release, thereby restoring the Th1/Th2 and Th17/Treg balance, enhancing antioxidant capacity, and protecting the intestinal barrier [148]. PDA-coated berberine nanoparticles (PDA@BBR NPs) were prepared through the oxidative polymerization of dopamine under weakly alkaline conditions. The PDA coating extended colonic retention via significant adhesion, facilitated ROS-responsive drug release through oxidative degradation in the inflamed microenvironment, and allowed for adjustable release kinetics by adjusting the coating thickness [149]. In another study, a ROS-responsive polymer (CMC-Se) containing diselenide bonds was synthesized and employed to encapsulate curcumin, resulting in the formation of CUR@CMC-Se nanoparticles. The negatively charged surface facilitated electrostatic adhesion to the inflamed colonic mucosa. Meanwhile, the oxidation of diselenide bonds under high ROS levels triggered the specific release of curcumin. Moreover, the overexpressed folate receptors on oxidative-stressed colonic epithelial cells mediated the active endocytosis of the nanoparticles [150]. ROS-responsive NDDS can enhance treatment efficacy by ensuring the precise release of drugs within the inflamed colon. ROS-responsive NDDS can enhance treatment efficacy by ensuring the precise release of drugs within the inflamed colon.

### 3.2. pH-Sensitive NDDS

Along the gastrointestinal tract, notable pH gradients are present: the stomach exhibits an acidic environment, while the pH in the proximal and distal colon ranges from 5.7 to 7.2, a range that expands under UC conditions [151]. Based on this characteristic, pH-responsive NDDS can safeguard drugs from being released in the stomach and small intestine, and trigger drug release upon reaching the colon as a result of the pH change. For example, Li, W. et al. conjugated soy protein isolate (SPI) with polyguluronate (PG) through the Maillard reaction to obtain a glycoprotein (SPI-PG) for encapsulating resveratrol. The grafting of PG provided a steric hindrance layer of a certain thickness to prevent the adsorption and hydrolysis of protease in digestive solution. Meanwhile, the change in intestinal pH triggered the release of resveratrol by altering the structure and solubility of SPI, thus enhancing its bioavailability [152].

### 3.3. Electrostatic Adsorption/Enzyme-Responsive NDDS

Positively charged proteins, including transferrin and antimicrobial peptides, accumulate at intestinal inflammatory sites [153]. This characteristic allows negatively charged TCM delivery systems to adhere electrostatically to the inflamed mucosa and extend local retention. Disruption of gut microbiota homeostasis subsequently impacts the secretion and activity levels of various enzymes, which can be utilized to construct enzyme-responsive NDDS, such as azoreductase, cellulase, and α-amylase [154,155,156,157]. A prevailing trend in recent research has been the combination of enzyme-responsive strategies with ROS-responsive or electrostatic adhesion approaches to achieve stepwise and precise drug delivery to inflamed colonic sites. For instance, PAPE-SA, produced by reacting succinic anhydride (SA) with 4-(hydroxymethyl)phenylboronic acid (PAPE), was conjugated with β-cyclodextrin (CD) through an amide reaction to yield the dual-sensitive amphiphilic polymer PAPE-SA-CD. This polymer was then used to load celastrol into Cel/NPs. Cel/NPs possessed a negatively charged surface, endowing them with favorable colon-targeting capability. Meanwhile, the high local ROS concentration in the colon triggered PAPE cleavage, and the β-CD component responded to α-amylase, jointly enabling precise and rapidly responsive drug release. Moreover, Cel/NPs circumvented the safety issue of celastrol-induced hepatotoxicity [158]. Another study employed electrostatic layer-by-layer self-assembly to alternately deposit cationic trimethyl chitosan (TMC) and anionic pectin onto celastrol-loaded core liposomes, constructing oral colon-targeted celastrol layer-by-layer coated liposomes (Cel/PT-LbL Lipo). These negatively charged particles were anchored on the surface of inflamed mucosa, and then pectin was enzymatically degraded by the microbiota, exposing positively charged TMC liposomes. This delivery strategy enhanced mucosal adhesion, cellular uptake, and tissue penetration while significantly reducing toxic side effects [159]. Both strategies effectively addressed the drawbacks of celastrol, namely its poor water solubility, gastrointestinal instability, toxicity, and insufficient colon targeting.

### 3.4. NDDS Based on Receptor-Ligand Specific Recognition

To further enhance the selectivity of drug accumulation in the lesion area, researchers have developed NDDS based on receptor–ligand specific recognition. Modifying the surface of nanocarriers with targeting ligands enables the precise recognition of overexpressed receptors at the inflamed site. Current research on receptor-targeted NDDS for TCM active ingredients primarily focuses on three targets: CD44, mannose receptor, and folate receptor.

Hyaluronic acid (HA) has been commonly utilized as ligands in the design of CD44-targeted NDDS. Lv, F. et al. developed HA-coated nanoparticles encapsulating an apigenin-Mn (II) complex (API-Mn (II)@HA NPs). These nanoparticles achieved active targeting through HA encapsulation, while Mn^2+^ synergistically scavenged ROS [160]. Lei, H. et al. constructed double-coated metal-organic framework (MOF) nanoparticles (Cur@MOF@HA-PDA NPs) for colon-targeted curcumin delivery. Aminated UiO-66 was synthesized with Zr as the metal center and 2-aminoterephthalic acid as the ligand. Curcumin was effectively loaded into its porous structure, followed by coating with HA and PDA sequentially. Here, PDA provided gastric resistance, and ROS-triggered degradation exposed HA for CD44-mediated macrophage targeting [161].

Mannose modification is regularly employed to confer mannose receptor-targeting capability to NDDS. Zhao, Y. et al. prepared celastrol-loaded nanoparticles (MC@Cel-NPs) by dissolving celastrol and PLGA in dichloromethane as the oil phase, and then emulsifying it with an aqueous phase containing chitosan and mannose. MC@Cel-NPs demonstrated an increased negative surface charge, enabling electrostatic adsorption-based targeting to the inflamed mucosa. Simultaneously, the mannose moiety facilitated macrophage uptake. The NPs significantly reduced the hepatotoxicity and hemolytic toxicity related to free celastrol [162]. Moreover, Han, X. et al. designed a multi-stage delivery system (Man-CUR NYPs) in which curcumin-loaded, mannose-modified pH/ROS dual-sensitive nanoparticles are encapsulated within yeast microparticles (YPs). In the colon, β-glucanase triggered YPs degradation to release the nanoparticles, which then target macrophage mannose receptors for cellular uptake and pH/ROS-responsive curcumin release [163].

Folate receptor-targeted delivery is generally accomplished through the modification of the nanocarrier with folic acid (FA). Ye, N. et al. designed an oral dual-targeted NDDS in which lactoferrin (LF) functioned as both the carrier and the targeting ligand. After conjugating FA to LF and loading curcumin, the resultant FA/CUR nanoparticles were coated with laminarin. This system realized colonic microbiota-triggered release and dual active targeting through the binding of LF to LF receptors on intestinal epithelial cells and the binding of FA to folate receptor on inflammatory macrophages [164]. Separately, Wu, M. et al. encapsulated FA-modified curcumin-loaded long-circulating liposomes (FA/CUR-PEG-LPs) within a pectin-chitosan (PC) hydrogel to construct a FA/CUR-PEG-LPs@PC composite formulation. The FA ligands exposed on the liposome surface specifically recognized and bound to the overexpressed folate receptor on inflammatory macrophages, thus promoting active cellular uptake [165].

Additionally, sialic acid-modified oxymatrine nanoparticles (OMT/SA-NPs) have been demonstrated to bind to the overexpressed Siglec-9 receptors on macrophages, thereby mediating the efficient uptake of nanoparticles [166]. Berberine-loaded β-glucan nanoparticles (GLC/BER) utilized the specific recognition of β-glucan by the pattern recognition receptors on the macrophage surface (e.g., Dectin-1) to facilitate the efficient uptake of berberine by inflammatory macrophages [167].

In summary, carrier-based NDDS effectively address the pharmacokinetic defects of TCM active ingredients by responding to specific signals of the UC pathological microenvironment, thereby laying a solid foundation for clinical translation. In the future, smart delivery systems integrating multiple responsive mechanisms and active targeting strategies will represent the development direction in this field. Furthermore, most current designs of NDDS rely on single- or dual-responsive mechanisms, whereas the heterogeneous and dynamic nature of the UC microenvironment necessitates more rigorous validation of multi-responsive systems.

## 4. Co-Delivery NDDS for TCM Ingredients

The multifaceted pathogenesis of UC, which encompasses inflammatory responses, oxidative stress, immune dysregulation, and gut microbiota dysbiosis, presents a challenge that a single active ingredient is insufficient to address. Motivated by the theory of “compatibility and synergy” in TCM, recent endeavors have concentrated on the construction of co-delivery NDDS. These systems allow for the simultaneous loading of two or more synergistic therapeutic components, thereby achieving multi-component co-delivery for UC treatment.

### 4.1. Co-Delivery NDDS of TCM Ingredients

The co-loading of two or more synergistic TCM active ingredients into nanocarriers has led to the development of diverse co-delivery systems, including polymer nanoparticles, layer-by-layer self-assembled nanoparticles, and hydrogels. Li, Y. et al. prepared oral Zein-CS-Cur-Que nanoparticles that co-load curcumin and quercetin using zein and sodium caseinate. Quercetin promoted the colonic absorption and retention of curcumin by efflux transporters and inhibiting intestinal metabolic enzymes. Meanwhile, curcumin and quercetin synergistically exerted anti-inflammatory, antioxidant, gut microbiota-modulating, and SCFA-promoting effects [168]. Liu, K. et al. constructed a Que-HQP-EGCG-CLRS micellar co-delivery system using hydrolytic quinoa protein (HQP) as an amphiphilic carrier. Quercetin was encapsulated in the hydrophobic core, with hydrophilic epigallocatechin 3-gallate (EGCG) adsorbed onto the surface and cationic lotus root starch (CLRS) serving as the outer layer. HQP and CLRS had the ability to protect Que and EGCG from rapid digestion in the stomach and ensure prolonged colonic retention. As a result, Que-HQP-EGCG-CLRS exerted anti-inflammatory and antioxidant effects, demonstrating superior efficacy compared to free drugs [18]. Yang, R. et al. devised a pH-responsive dual-delivery system (Cur/GA-micelle-gel-beads), in which glycyrrhizic acid micelles loaded with curcumin were incorporated into the sodium alginate-chitosan composite hydrogel. This system demonstrated favorable colon-targeting properties and anti-UC efficacy [19]. Multi-component delivery can be accomplished using plant-derived vesicles. For instance, a supramolecular hydrogel (Kae/CMCHD@RNs), constructed from a furfural-functionalized chitosan-mannose polymer and synthesized 3-maleimide hydroxypropyl-β-cyclodextrin for the colonic co-delivery of rhubarb-derived nanovesicles (RNs) and kaempferol, targeted macrophage mannose receptors and utilized pH/enzyme sensitivity for sustained release. This enabled RNs (enriched in rhein, emodin, and chrysophanol) and kaempferol to synergistically alleviate the symptoms of UC [169]. Additionally, the application of degradable polysaccharide coatings on NDDS presents an approach for co-delivery. Tao, C. et al. developed Ba@MP/LBP NPs by first mannosylating peach kernel protein (PKP) via the Maillard reaction, then loading the mannosylated PKP with baicalin, and finally coating it with anionic *Lycium barbarum* polysaccharide. After reaching the colon, the outer shell of polysaccharide was hydrolyzed by microbial enzymes, then the exposed Ba@MP NPs were specifically taken up by inflammatory macrophages through mannose receptor-mediated endocytosis. *Lycium barbarum* polysaccharide synergistically enhanced the anti-inflammatory and intestinal barrier repair effects of baicalin [170].

### 4.2. Co-Delivery NDDS of TCM Active Ingredients and Gas Molecules

Increasing attention has been paid to the essential functions of gasotransmitters, such as nitric oxide (NO) and carbon monoxide (CO), in promoting the resolution of inflammation and protecting tissues. Considering their mechanistic complementarity and synergy with the active ingredients of TCM, the co-delivery of NDDS for TCM active ingredients and gas molecules represents an emerging paradigm.

NO possesses both pro-inflammatory and anti-inflammatory properties. Excessive amounts of iNOS-derived NO worsen tissue damage. In contrast, exogenous low-dose sustained-release NO exhibits anti-inflammatory and tissue-protective properties [171,172,173]. A study constructed a liposome system (OM@TN-lip) for the co-delivery of oxymatrine and NO, achieving synergistic delivery of the two therapeutics by embedding the NO donor d-α-tocopheryl polyethylene glycol succinate nitrate (TPGS nitrate, TN) into the liposomal system while encapsulating oxymatrine. Negatively charged liposomes could achieve electrostatic adsorption to the positively charged inflamed colonic mucosa for passive targeting, thereby achieving synergistic effects for the treatment of UC. This effect is reflected in the superior ability of OM@TN-lip to downregulate TNF-α, IL-1β, IL-6, and IFN-γ, as well as to reduce ROS levels and elevate GSH levels, compared to OM@lip or TN+OM [171]. CO is predominantly produced through the heme oxygenase (HO)-mediated heme metabolism, establishing a regulatory loop with HO-1 to exert anti-inflammatory, antioxidant, and tissue-protective effects [174,175]. The responsive release of CO in the colitis microenvironment reverses the pro-inflammatory state and restores the intestinal barrier function [176]. A co-delivery system for TCM active ingredients and CO (AG/CORM-2@NP-Dex) was developed to co-encapsulate andrographolide and the CO donor CORM-2, modified with dextran (Dex) to endow active targeting ability, and then encapsulated in a chitosan/alginate hydrogel. The hydrogel prevented release in the gastric environment while facilitating release in the colonic environment. CO was released in a sustained manner for more than 12 h, synergizing with andrographolide to achieve anti-inflammatory and pro-resolving treatment [177].

The co-delivery NDDS of TCM active ingredients not only reflects the traditional theory of “compatibility and synergy” in TCM but also offers a novel approach for the treatment of complex diseases. Through the rational design of drug combinations and delivery carriers, the spatiotemporal synergy of therapeutic agents can be realized, enhancing the overall efficacy while reducing the dosage and minimizing adverse effects. However, the in vivo fate of these co-delivery systems within the inflamed colonic microenvironment—including the biodistribution, release kinetics, and long-term safety—remains insufficiently investigated, warranting systematic evaluation prior to clinical translation.

## 5. Carrier-Free NDDS for TCM Active Ingredients

Conventional NDDS rely on synthetic carrier materials (such as PLGA, liposomes, and PEG.) and are confronted with issues such as low drug loading capacity, burst release, and potential carrier toxicity [178,179,180]. Carrier-free NDDS, which do not require synthetic carriers, effectively enhance the pharmacokinetic and pharmacodynamic properties of TCM active ingredients, optimize the pharmacological activity, and reduce adverse reactions [20,181,182]. The self-assembly and bio-derived vesicle delivery are significant application forms of carrier-free NDDS in UC therapy of TCM active ingredients.

Certain active ingredients of TCM are capable of constructing self-assembled systems via non-covalent interactions, including hydrogen bonding, van der Waals forces, π–π stacking, and electrostatic interactions. These self-assembled systems not only improve the pharmacokinetic properties but also enhance the therapeutic efficacy through intermolecular synergy [183,184]. For example, berberine and magnolol were utilized to drive carrier-free self-assembly through electrostatic interactions and π–π stacking, resulting in the formation of nano-assemblies named BM. These BM nano-assemblies promoted endocytosis by intestinal epithelial cells. Additionally, magnolol inhibited P-glycoprotein efflux, thereby enhancing the colonic accumulation of berberine. BM demonstrated slow release under acidic gastric conditions and achieved sustained release in the near-neutral pH environment of the colon, leading to enhanced anti-inflammatory, barrier-repairing, and gut microbiota-modulating effects [185]. Berberine could also self-assemble with hesperetin under alkaline conditions, driven by electrostatic interactions, π–π stacking, and hydrogen bonding, to spontaneously form carrier-free BBR-HST NPs. Relying on passive targeting, BBR-HST NPs exhibited sustained release profiles in both gastric acid and colonic neutral environments, and exerted anti-inflammatory effects that were superior to those of the free monomers [183]. Moreover, berberine and tannic acid can spontaneously assemble through hydrogen bonds and π–π stacking interactions to form spherical nanoparticles (TB) [186]. A study successfully isolated naturally occurring submicron precipitates from the aqueous decoction of Qingchang Wenzhong Decoction. These precipitates were identified as stable complexes that spontaneously assembled during the decoction process through non-covalent interactions. The complexes were primarily composed of berberine, matrine, and glycyrrhizic acid, and mediated by acid-base electrostatic interactions. Both complexes exhibit stronger anti-inflammatory effects compared to the free alkaloids [187].

Extracellular vesicles (EVs), encompassing mammalian cell-derived exosomes, microvesicles, and plant-derived nanovesicles, are commonly used natural biogenic carrier systems for TCM active ingredients. This is attributed to their natural lipid bilayer membrane structure, remarkable biocompatibility, low immunogenicity, and inherent targeting ability [20]. EVs are capable of encapsulating TCM active ingredients, safeguarding them from gastrointestinal degradation, and facilitating uptake by immune or epithelial cells at colonic inflammatory sites through surface protein- or lipid-mediated targeting. Huang, S. et al. employed PEG 8000 and sucrose density gradient ultracentrifugation to extract ginger-derived nanovesicles (GDNVs), and subsequently loaded curcumin to form CG nanovesicles, which exhibited good stability and sustained release in colon [188]. Likewise, turmeric-derived nanoparticles (TDNPs 2) were isolated via ultracentrifugation and sucrose density gradient purification. Composed of lipids, proteins, and curcumin, TDNPs 2 preferentially accumulated at inflamed colonic sites upon oral administration. They were internalized by colonic epithelial cells and macrophages, inhibited NF-κB activation, reduced pro-inflammatory cytokines (TNF-α, IL-6, IL-1β), upregulated HO-1, and promoted wound healing and inflammation resolution [189]. Deng, C. et al. fabricated Exos-Ber, which exhibited favorable stability and sustained release properties, by co-incubating berberine with exosomes derived from human placental mesenchymal stem cells. Following intravenous injection, it targeted the inflamed colon tissue, demonstrating superior therapeutic efficacy compared to free berberine [190].

These carrier-free NDDS based on TCM active ingredients have created new opportunities for attaining precise, efficient, and low-toxicity treatment of UC. Nevertheless, the in vivo fate of these systems within the inflamed colonic microenvironment remains unexplored, which represents a critical direction for the further development of carrier-free nanodrug delivery strategies for active ingredients of TCM.

## 6. Prodrug Design

Prodrug design via chemical modification of TCM active ingredients, which directly modifies the physicochemical properties (e.g., solubility, lipophilicity, and stability), represents another crucial strategy for enhancing the druggability of TCM. Through esterification, salt formation, or oxidation, prodrug design improves pharmacokinetics and bioavailability, thus enhancing therapeutic efficacy. For instance, baicalin magnesium, a natural component derived from decoction, demonstrated superior anti-UC effects compared to baicalin or magnesium sulfate alone by modulating gut microbiota and bile acid metabolism, suppressing the PPARα/NF-κB pathway [191]. Guo, M. et al. synthesized baicalin n-butyl ester (BNE) by modifying the 6-carboxyl group of baicalin with n-butanol through an esterification reaction. BNE exhibited remarkable therapeutic effects by directly binding to the ERK protein, suppressing the ROS/ERK/p-ERK/NLRP3 signaling pathway, inhibiting pyroptosis, and regulating the structure of the gut microbiota [192]. Additionally, Li, C. et al. synthesized oxyberberine using potassium ferricyanide and berberine in 20% sodium hydroxide. By converting the quaternary ammonium nitrogen at the C8 position to enhance its biological activity, oxyberberine inhibited inflammatory response and oxidative stress by acting on the Keap1/Nrf2/NF-κB pathway, showing significantly better efficacy than berberine [193,194]. Similarly, the prodrug design of such active ingredients from TCM necessitates a clearer elucidation of the disposition to avoid compromising the activation specificity of the prodrug within the gut.

## 7. Challenges and Perspectives of TCM Active Ingredients in UC Treatment

### 7.1. Unraveling the Multi-Level Regulatory Network of TCM Active Ingredients

As presented in Table 1, the majority of TCM active ingredients demonstrate multi-level regulatory characteristics. The five categories of TCM active ingredients, namely flavonoids, alkaloids, phenols, terpenoids, and polysaccharides, predominantly exert anti-UC effects via multi-level mechanisms. These mechanisms encompass the modulation of inflammatory signaling pathways (such as NF-κB, PI3K-AKT, NLRP3, and JAK/STAT), the repair of the intestinal mucosal barrier (through the upregulation of ZO-1, Occludin, and Claudin), the regulation of gut microbiota and metabolites (including SCFAs bile acids, tryptophan, and tyrosine), and the restoration of immune cell homeostasis, which mainly includes the Th17/Treg balance and M1/M2 macrophage polarization. Nevertheless, the crosstalk and mechanisms within the interaction network have not been systematically deconstructed. Moreover, the rapid metabolism and complex metabolic fate of these active ingredients, along with the insufficiency of pharmacokinetic-pharmacodynamic correlation studies, hinder the identification of the effective forms and action targets in vivo. Hence, future research should integrate chemical proteomics, single-cell sequencing, spatial metabolomics, and in vivo imaging techniques to systematically clarify the action targets, pharmacokinetic behaviors, and dose–response relationships of TCM active ingredients under complex pathological conditions, thereby constructing a comprehensive multi-target regulatory network.

### 7.2. Chemoproteomics-Driven Target Identification of TCM Active Ingredients

An increasing number of studies have utilized molecular docking, biotinylated probes, ABPP, and other chemoproteomic techniques to identify and investigate the direct targets of TCM active ingredients in the treatment of UC. Further verification of direct molecule-protein interactions is carried out through assays such as CETSA and SPR, in combination with gene knockdown/knockout or pharmacological intervention to confirm the functional necessity of the targets, offering direct evidence for clarifying the “precise mechanisms” of TCM active ingredients. Nevertheless, the majority of existing target studies have been conducted in systems lacking an intestinal inflammatory background, and consequently lack in situ assessment of target binding activity and conformational changes under UC pathological conditions (e.g., inflammatory cytokine stimulation and oxidative stress). Moreover, since a single active ingredient may act on multiple proteins, precisely dissecting the cascade regulatory effects triggered by these target proteins remains a crucial research direction for advancing precision therapeutic strategies based on TCM ingredients. Therefore, future research should develop target validation technologies based on in situ molecular imaging, gene editing, and organoid models to achieve real-time dynamic monitoring of drug–target interactions within the UC pathological microenvironment, providing a direct foundation for precision therapy.

### 7.3. Bridging Structural Changes, Metabolic Enzymes, and Host Interactions

Numerous studies have verified that a variety of active ingredients in TCM can indirectly improve UC by regulating gut microbiota homeostasis and metabolic activity levels. Nevertheless, current research has mainly focused on characterizing the structural changes in the gut microbiota induced by TCM active ingredients through 16S rRNA or metagenomic methods. However, there is still a dearth of in-depth exploration regarding how these ingredients influence specific bacterial functions or their mechanisms of interaction with the host. Although certain studies have examined the regulation of various metabolite profiles, such as SCFAs, bile acids, tryptophan, and tyrosine, the structure–activity relationships between TCM active ingredients and gut microbial metabolic enzymes remain ambiguous. Moreover, whether the modulation of microbial metabolic activity stems from a direct action on the microbiota or an indirect intervention in metabolic enzymes or pathways requires further clarification. These knowledge gaps necessitate an integrated approach employing metagenomics, metabolomics, and FMT to identify crucial functional bacteria and elucidate the core mediating functions of metabolic pathways. Simultaneously, applying synthetic biology and enzymology to clarify the structure–function relationships between TCM active ingredients and gut microbial metabolic enzymes will support the development of drug delivery strategies guided by microbiota metabolism.

### 7.4. Overcoming Pharmacokinetic Bottlenecks and Carrier Limitations

Pharmacokinetic bottlenecks have consistently presented a non-negligible challenge to the druggability of TCM active ingredients. Research on novel delivery strategy provides a viable approach for their clinical translation. Recent progress in NDDS has enabled colon-targeted delivery and controlled release. Nevertheless, the majority of these NDDS employ single-signal responsive mechanisms. Considering the complex and variable microenvironment of UC, along with inter-individual differences in gut microbiota and heterogeneity in inflammation severity, these factors may reduce the precision of drug release. Moreover, owing to unknown cumulative toxicity and metabolic pathways, the long-term biosafety of certain carriers (e.g., metal–organic frameworks), as well as whether they undergo other modification reactions or sudden release in vivo, remain uncertain, which impedes clinical translation [195,196,197].

The self-assembly strategies of TCM active ingredients overcome the limitations of conventional carriers by preserving the pharmacological activity of each component and achieving synergistic effects through molecular-level assembly [185,198]. Current research still encounters numerous challenges. The self-assembly mechanisms of TCM active ingredients are based on the qualitative characterization of non-covalent interactions, including hydrogen bonding, π–π stacking, and electrostatic interactions. However, there is still a lack of systematic analysis of the thermodynamics and kinetics of the assembly process. The assembly driving forces vary significantly among different ingredients, making it difficult to establish a universal predictive model. Additionally, the in vivo dynamic behavior (absorption, transport, distribution, clearance) of self-assembled nanoparticles is still poorly understood [198]. Most self-assembled nanoparticles rely on size-mediated passive accumulation, whereas active targeting design for the colonic inflammatory microenvironment in UC remains relatively underexplored. Future research ought to utilize multi-scale characterization techniques in combination with thermodynamic parameter analysis and kinetic analysis, along with machine learning models to forecast assembly behavior, thereby offering theoretical guidance for a more universal design. Furthermore, pH-responsive, enzyme-responsive, ROS-responsive, and targeting ligand modification strategies should be organically integrated with self-assembly technology to construct multi-dimensional, multi-level smart NDDS that can achieve “hierarchical precision delivery”. Additionally, multi-omics and in vivo imaging techniques should be exploited to further clarify the in vivo fate of self-assemblies.

## 8. Conclusions

This article conducts a systematic review of the research progress regarding TCM active ingredients for the treatment of UC over the past five years. It comprehensively showcases the latest achievements from three aspects: chemical diversity, mechanisms of action, and druggability optimization. Research findings have indicated that TCM active ingredients demonstrate unique advantages in UC treatment through regulation involving multiple targets and pathways. Furthermore, the advancements in target validation technologies, in-depth explorations of the gut microbiota, and the application of novel NDDS are propelling the shift of TCM anti-UC research from empirical medicine towards precision medicine. Additionally, rigorous, large-scale, randomized controlled clinical trials are essential to validate the efficacy and safety of various TCM active ingredients and NDDS. Further, stratifying patients based on gut microbiota composition, inflammatory endotypes, or pharmacogenomic markers may enable personalized TCM therapy.

**Figure 7:**
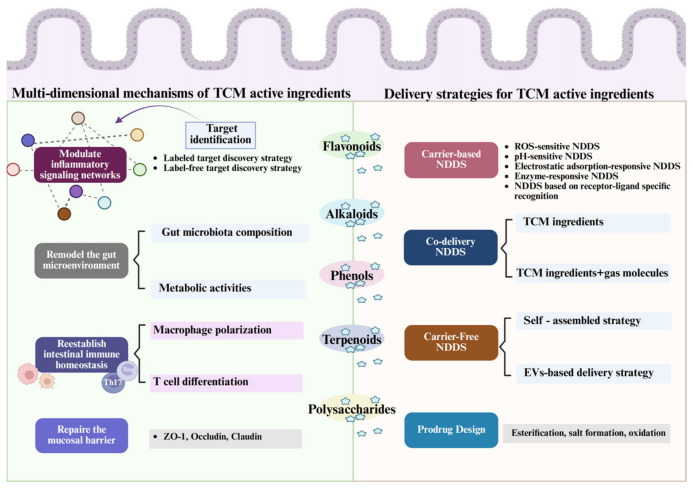
A retrospective summary of research on TCM active ingredients for UC treatment. Created in BioRender. Fan, Q. (2026) (https://BioRender.com/w358spj, accessed on 27 May 2026).

In summary, the active ingredients of TCM present extensive application prospects in anti-UC therapy, and further progress in this field depends on the multi-mechanism effects and the development of drug delivery strategies aimed at clinical translation (Figure 7). As the mechanistic studies continue to deepen and translational technologies keep innovating, TCM is expected to play an even more crucial role in the prevention and treatment of UC, offering Chinese wisdom and solutions to the global management of UC.

## Figures and Tables

**Table 1:** Summary of pharmacodynamic effects of TCM active ingredients in the treatment of UC.

Types	Compounds	Source	Experimental Models	Efficacy Indicators and Mechanisms	Study Type	Ref.
Flavonoids	Baicalin	*Scutellaria baicalensis* Georgi	3% DSS-induced BALB/c mice; Th-macrophages co-culture system	↓ weight loss, ↓ colon shortening, ↓ DAI; ↓ TNF-α, ↓ IL-1β, ↓ IL-6, ↓ IFN-γ, ↓ IL-23; inhibited JAK2/STAT3 and Th17 cells differentiation, inhibited NF-κB and M1 macrophage polarization	In vivo and In vitro	[28]
			3% DSS-induced C57BL/6 mice; 1 μg/mL LPS-induced RAW264.7 cells	↓ weight loss, ↓ colon shortening, ↓ DAI; inhibited CD14/TLR4/NF-κB and activated PPAR-γ	In vivo and In vitro	[48]
	Baicalein	*Scutellaria baicalensis* Georgi	3% DSS-induced C57BL/6 mice; MNK-3 cells	↓ weight loss, ↓ colon shortening, ↓ DAI; ↑ ZO-1 and Occludin, activated AhR/IL-22 pathway	In vivo and In vitro	[24]
			4% DSS-induced C57BL/6 mice	↓ weight loss, ↓ colon shortening, ↓ DAI; ↓ TNF-α, ↓ IL-1β, ↓ ROS, ↓ MDA; ↑ IL-10, ↑ SOD, ↑ MPO, ↑ GSH; inhibited ferroptosis and regulated gut microbiota	In vivo	[49]
			5% DSS-induced *C. elegans* model	↑ lifespan, ↑ body lenth, ↑ body width, ↓ ROS, ↓ MDA, ↑ SOD, ↑ head swings, ↑ body bends; restored DAF-16 nuclear translocation; activated p38 MAPK and IIS	In vivo	[50]
	Quercetin	*Sophora japonica* L., *Portulaca oleracea* L.	3% DSS-induced C57BL/6 mice; RAW264.7 cells and BMDMs	↓ weight loss, ↓ colon shortening, ↓ DAI; ↓ CXCL10, ↓ TNF-α, ↑ IL-10, ↑ CCL17; ↑ ZO-1 and Occludin; inhibited cGAS-STING and restored M2/M1 balance	In vivo and In vitro	[51]
			3% DSS-induced C57BL/6mice; THP1 supernatant-induced Caco-2 cells	↓ weight loss, ↓ colon shortening, ↓ DAI; ↑ ZO-1 and Claudin-1; activated AhR	In vivo and In vitro	[23]
			2% DSS-induced C57BL/6N mice; LPS or PMA-induced neutrophils	↓ weight loss, ↓ colon shortening, ↓ DAI; ↑ ZO-1, Claudin-1 and Occludin; ↓ TNF-α, ↓ IL-6, ↓ IFN-γ, ↓ ROS; inhibited NF-κB and activated AhR/Arnt and Nqo1	In vivo and In vitro	[52]
			3% DSS-induced C57BL/6 mice	↓ weight loss, ↓ colon shortening, ↓ DAI; ↓ TNF-α, ↓ IL-1β, ↑ ZO-1, MUC2 and Occludin; inhibited CXCL8-CXCR1/2	In vivo	[53]
			3% DSS-induced C57BL/6 mice	↓ weight loss, ↓ colon shortening, ↓ DAI; ↓ TNF-α, ↓ IL-1β, ↓ IL-6; ↑ Occludin and Claudin-1; modulated gut bacteria and tryptophan metabolism	In vivo	[54]
	Luteolin	*Lonicera japonica* Thunb.	3.5%DSS-induced Wistar rats	↓ DAI; ↓ IL-17, ↓ IL-23; modulated gut bacteria	In vivo	[55]
			3% DSS-induced C57BL/6 mice; 1 μg/mL LPS-induced RAW264.7 cells and primary peritoneal macrophages	↓ weight loss, ↓ colon shortening, ↓ DAI; ↑ ZO-1 and Occludin; ↓ TNF-α, ↓ IL-6, ↓ IL-1β; inhibited IKKα/β and NF-κB	In vivo and In vitro	[30]
			3%DSS-induced BALB/c mice; Tunicamycin and Thapsigargin-induced HT-29 cells	↓ weight loss, ↓ colon shortening, ↓ DAI; ↓ TNF-α, ↓ IL-6, ↓ IL-1β; ↑ ZO-1, Claudin-1 and Occludin; modulated gut bacteria; inhibited ER stress and apoptosis by targeting SIRT1	In vivo and In vitro	[56]
			3%DSS-induced BALB/c mice; 1 μg/mL LPS-induced RAW264.7 cells	↓ weight loss, ↓ colon shortening, ↓ DAI; ↓ TNF-α, ↓ IL-6, ↓ IL-1β, ↑ IL-10; ↑ ZO-1, Claudin-4 and Occludin; reestablished the M1/M2 macrophages polarization balance by AMPK–PPARγ pathway	In vivo and In vitro	[57]
			3% DSS-induced C57BL/6J mice; 250 ng/mL LPS-induced THP-1 cells; 250 ng/mL LPS-induced BMDMs	↓ weight loss, ↓ colon shortening, ↓ DAI; ↓ MPO, ↓ IL-1β; ↑ ZO-1, Claudin-1, MUC2 and Occludin; ↓ ROS, ↓ mtROS, ↓ Ca^2+^; activated AMPK and inhibited NLRP3	In vivo and In vitro	[34]
			3%DSS-induced BALB/c mice	↓ weight loss, ↓ colon shortening, ↓ DAI; ↓ TNF-α, ↓ IL-6, ↓ IL-1β, ↑ IL-10; ↑ ZO-1, Claudin-1 and Occludin; modulated gut bacteria and plasma metabolism	In vivo	[58]
			2%DSS-induced C57BL/6J mice; MNK-3 cells	↓ weight loss, ↓ colon shortening, ↓ DAI; ↓ TNF-α, ↓ IL-6, ↓ IL-1β; ↑ ZO-1 and Occludin; ↑ NCR^+^ILC3; regulated Notch	In vivo and In vitro	[59]
			4%DSS-induced C57BL/6J mice; 25 ng/mL TNF-α and 25 ng/mL IFN-γ-induced Caco-2 cells	↓ weight loss, ↓ colon shortening, ↓ DAI; ↑ ZO-1, Claudin-1, Claudin-2 and Occludin; regulated SHP-1/STAT3	In vivo and In vitro	[60]
	Kaempferol	*Ginkgo biloba* L.	3.5% DSS-induced C57BL/6J mice	↓ colon shortening, ↓ DAI; ↓ TNF-α, ↓ IL-6, ↓ IL-1β; ↑ ZO-1, Claudin-1 and Occludin; modulated gut bacteria; inhibited TLR4-NF-κB	In vivo	[29]
			2.5% DSS-induced C57BL/6J mice; 500 ng/mL LPS and 2 mM ATP-induced peritoneal macrophages	↓ weight loss, ↓ colon shortening; ↓ IL-6, ↓ IL-1β; activated Nrf2 and inhibited NLRP3 inflammasome	In vivo and In vitro	[33]
	Apigenin	*Plantago asiatica* L.	3% DSS-induced C57BL/6 mice; LPS-induced RAW264.7 cells; BMDMs	↓ weight loss, ↓ colon shortening, ↓ DAI; ↓ IL-6, ↓ IL-1β, ↓ CCL2, ↓ NO; ↑ MUC2 and Occludin; modulatd IRAK4, NF-κB and MAPK	In vivo and In vitro	[61]
			5%DSS-induced C57BL/6 mice and MC MrgprB2-CKO mice; PAMP-12-induced LAD2 cells; HEK293-MRGPRX2 and MPMC cells	↓ colon shortening, ↓ DAI; ↓ TNF-α, ↓ MPO, ↓ IL-8, ↓ MCP-1; inhibited mast cell degranulation by Akt1/XBP-1S/CHOP/TXNIP and NF-κB/IL-1β	In vivo and In vitro	[62]
			3%DSS-induced BALB/c mice; TM and TG-induced LS 174T goblet cells	↓ weight loss, ↓ colon shortening, ↓ DAI; ↑ ZO-1, Claudin-1 and Occludin; modulated gut bacteria; activated SERCA2 and inhibited PERK-eIF2a-ATF4-CHOP	In vivo and In vitro	[63]
			2.5%DSS-induced C57BL/6 mice; 50 ng/mL LPS-induced BMDMs; 100 ng/mL LPS-induced RAW264.7 cells	↓ weight loss, ↓ colon shortening, ↓ DAI; ↑ ZO-1 and Occludin; ↑ Bcl/Bax, ↓ MDA, ↑ GSH, ↑ T-AOC; ↓ TNF-α, ↓ IL-6, ↓ IL-1β; modulated AMPK/NF-κB/NLRP3	In vivo and In vitro	[31]
	Naringenin	*Citrus sinensis* (L.) Osbeck	3.5% DSS-induced BALB/c mice	↓ weight loss, ↓ colon shortening, ↓ DAI; ↓ TNF-α, ↓ IL-1β; activated Nrf2 and inhibited NF-κB	In vivo	[32]
	Hesperetin		2.5% DSS-induced C57BL/6 mice; LPS-induced RAW264.7 cells	↓ weight loss, ↓ colon shortening, ↓ DAI; ↓ TNF-α, ↓ IL-6; ↓ MDA, ↑ SOD; suppressed ferroptosis and modulated gut microbiota	In vivo and In vitro	[64]
	Genistein	*Pueraria lobata*(Willd.)Ohwi	5% Acetic acid-induced Wister rats	↓ weight loss, ↓ colon mass, ↓ DAI; ↓ TNF-α, ↓ IL-1β, ↓ IL-6; modulated INF-γ/JAK1/STAT1/IRF-1, TLR-4/NF-κB/IL-6, and JAK2/STAT3/COX-2 crosstalk	In vivo	[65]
			1% or 2% DSS-induced C57BL/6 mice	↓ weight loss, ↓ colon shortening; ↓ TNF-α, ↓ IL-6; ↑ MUC2, Claudin-1, Occludin and ZO-1; increased the relative abundance of *Marvinbryantia formexigens* and SCFAs; promoted COX-2	In vivo	[66]
Alkaloids	Berberine	*Coptis chinensis* Franch.	3% DSS-induced C57BL/6 mice; 50 µg/mL LPS-induced NCM460 cells	↓ weight loss, ↓ colon shortening, ↓ DAI; ↓ TNF-α, ↓ IL-6; inhibited TLR4/NF-κB/HIF-1α	In vivo and In vitro	[67]
			High-fat + High-sugar + 2% DSS-induced C57BL/6J mice; 1 µg/mL LPS-induced RAW264.7 cells	↓ colon shortening, ↓ DAI; ↓ TNF-α, ↓ MPO, ↓ IL-6, ↓ IL-1β; ↑ ZO-1, Claudin-1 and Occludin; inhibited PI3K/AKT/mTOR by targeting IRGM1	In vivo and In vitro	[68]
			3% DSS-induced C57BL/6 mice; 200 ng/mL LPS or 20 ng/mL IL-4-induced RAW 264.7 cells	↓ colon shortening, ↓ CMDI; ↓ CD11b^+^F4/80^+^CD86^+^cells, ↑ CD11b^+^F4/80^+^CD206^+^cells; activated IL-4-STAT6	In vivo and In vitro	[69]
			3% DSS-induced Balb/c mice; 50ng/mL TNF-α-induced Caco-2 cells	↓ weight loss, ↓ colon shortening, ↓ DAI; ↑ ZO-1 and Occludin; ↓ CD127^+^Lin^-^T-bet^+^ cells, ↑ CD127^+^Lin^-^ROR-γt^+^ cells; ↓ ROR-γt^+^CD4^+^Th17 cells, ↑ Foxp3^+^CD4^+^Treg cells; regulated gut microbiota; activated Wnt/β-catenin	In vivo and In vitro	[37]
			2.3% DSS-induced C57BL/6J mice; 20 ng/mL IL-4 and 20 ng/mL IL-13-induced RAW264.7 cells	↓ weight loss, ↓ colon shortening; ↓ TNF-α, ↓ IL-6, ↓ IL-1β; ↓ F4/80^+^CD86^+^ and ↑ F4/80^+^CD206^+^ macrophages; modulated PPAR-γ/mTOR/HIF-1α	In vivo and In vitro	[70]
			3% DSS-induced Balb/c mice	↓ weight loss, ↓ colon shortening, ↓ DAI; ↓ TNF-α, ↓ IL-1β; ↑ MUC2, MUC3 and Occludin; modulated gut microbiota, elevated unconjugated and secondary bile acids; activated FXR and TGR5	In vivo	[71]
	Matrine	*Sophora flavescens* Ait.	3% DSS-induced C57BL/6J mice	↓ weight loss, ↓ colon shortening, ↓ DAI; ↓ TNF-α, ↓ IL-6, ↓ IL-1β, ↓ IL-17A, ↑ IL-10, ↓ NO, ↓ MDA, ↑ SOD, ↑ T-AOC, ↑ CAT; ↑ ZO-1 and Occludin; regulated Treg/Th17 cells and modulated gut microbiota	In vivo	[38]
			3% DSS-induced C57BL/6J mice	↓ weight loss, ↓ colon shortening; ↓ TNF-α, ↓ IL-6, ↓ IL-1β; ↑ ZO-1, Claudin-1, Claudin-2 and Occludin; inhibited the PPAR-α and modulated gut microbiota	In vivo	[72]
			3% DSS-induced C57BL/6J mice; 200 ng/mL LPS-induced MODE-K cells	↓ weight loss, ↓ colon shortening, ↓ DAI; ↓ TNF-α, ↓ IL-6, ↓ IL-1β, ↓ NO, ↓ MDA, ↓ MPO; improved bile acid metabolism; inhibited JAK2/STAT3	In vivo and In vitro	[73]
	Oxymatrine	*Sophora flavescens* Ait.	4% DSS-induced ICR mice	↓ colon shortening, ↑ colonic villi length; ↓ TNF-α, ↓ IL-6, ↓ IL-1β; improved ferroptosis	In vivo	[74]
			High-fat + High-sugar + 1.5% DSS-induced C57BL/6J mice; HCT116 and Caco-2 cells stimulated with TNF-α and IFN-γ	↓ weight loss, ↓ colon shortening, ↓ DAI; ↓ TNF-α, ↓ IL-1β, ↓ IL-17A, ↓ IFN-γ, ↓ MDA, ↑ GSH; ↑ ZO-1, Claudin-1, and Occludin; inhibited caspase-8 and reduces Dsg2 cleavage	In vivo and In vitro	[75]
			35 mg kg^−1^ TNBS-induced SD rats; 10 μg/mL LPS and 3 mM ATP-induced primary rat peritoneal macrophages; LPS/ATP-induced RAW264.7 cells	↓ CMDI, ↓ colon shortening, ↓ DAI; inhibited NLRP3, caspase-1, and GSDMD	In vivo and In vitro	[76]
	Sinomenine	*Sinomenium acutum* (Thunb.) Rehd. et Wils.	2% DSS-induced Swiss albino rats	↓ weight loss, ↓ colon shortening, ↓ DAI; ↓ NO, ↓ MPO; ↓ ICAM-1, ↓ VCAM-1; ↑ SOD, ↑ CAT, ↑ GPX, ↑ GR, ↓ MDA; ↓ TNF-α, ↓ IL-1, ↓ IL-1β, ↓ IL-2, ↓ IL-6, ↓ IL-17, ↓ IL-18, ↑ IL-10; ↑ HO-1, ↑ Nrf2; ↓ PAF, COX-2, PGE2, iNOS, NF-κB, MCP-1, MIP-2, TXB2, and LCT4; modulated gut microbiota	In vivo	[77]
			3% DSS-induced C57BL/6 mice; 0.8 mg/mL DSS-induced HCoEpiC; RAW264.7 cells	↓ colon shortening; ↓ TNF-α, ↓ IL-6, ↓ IL-1β, ↓ NO; elevated 14-3-3θ and inhibited NF-κB	In vivo and In vitro	[78]
	Palmatine	*Coptis chinensis* Franch., *Corydalis yanhusuo* W. T. Wang	5% DSS-induced SD rats; 2% DSS-induced NCM460 cells	↓ weight loss, ↓ colon shortening, ↓ DAI; ↓ TNF-α, ↓ IL-6, ↓ IL-1β, ↓ IL-18, ↓ LDH, ↓ MDA, ↓ NO, ↑ GSH; suppressed inflammatory responses, oxidative stress, and iron accumulation	In vivo and In vitro	[79]
			5% DSS-induced SD rats; 2% DSS-induced NCM460 cells	↓ weight loss, ↓ colon shortening, ↓ DAI; ↓ TNF-α, ↓ IL-6, ↓ IL-1β, ↓ IL-8; ↑ ZO-1; enhanced METTL3 and METTL14, decreased ALKBH5 and FTO	In vivo and In vitro	[80]
Phenols	Paeonol	*Paeonia suffruticosa* Andr., *Cynanchum paniculatum* (Bge.) Kitag.	3% DSS-induced C57BL/6 mice; 1 μg/mL LPS-stimulated RAW264.7 cells and NCM460 cells	↓ weight loss, ↓ colon shortening, ↓ DAI; ↓ TNF-α, ↓ IL-6, ↑ IL-10; ↓ ROS, promoted M2 macrophage polarization; enhanced PPARγ and inhibited NF-κB	In vivo and In vitro	[40]
			3% DSS-induced C57BL/6 mice	↓ weight loss, ↓ colon shortening, ↓ DAI; ↑ IL-22^+^ILC3, ↑ IL-22; ↑ ZO-1 and Occludin; increaed *C. butyricum* and SCFAs production	In vivo	[81]
			3% DSS-induced BALB/c mice	↓ weight loss, ↓ colon shortening, ↓ DAI; ↓ TNF-α, ↓ IL-6, ↓ IL-1β, ↑ IL-4; ↑ ZO-1 and Occludin; regulated gut microbiota, activated hepatic FXR-SHP/LRH-1 and ileal FXR-FGF15, restored bile acid metabolism and SCFAs	In vivo	[82]
	Rosmarinic acid	*Salvia miltiorrhiza* Bge.	3% DSS-induced ICR mice; 20 μg/mL LPS-stimulated NCM460 cells	↓ weight loss, ↓ colon shortening; ↓ DAI; ↑ ZO-1, Claudin-1 and Occludin; mediated PI3K/AKT/Nrf2	In vivo and In vitro	[42]
	Curcumin	*Curcuma longa* L., *Curcuma wenyujin* Y. H. Chen et C. Ling	2.5% DSS-induced BALB/c mice	↓ weight loss; ↓ TNF-α, ↓ IL-6, ↓ IL-1β, ↓ MPO; inhibited SphK1/NF-κB	In vivo	[39]
			1.5% DSS-induced C57BLKS/J (−/−) (DB) mice	↓ weight loss, ↓ colon shortening, ↓ DAI; ↓ CD4^+^CCR6^+^ and CD4^+^IL-17A^+^Th17 cells, ↑ CD4^+^Foxp3^+^ and CD4^+^IL-10^+^ Treg cells; improved the composition of intestinal microbiota; regulated Th17/Treg balance	In vivo	[83]
	Resveratrol	*Polygonum cuspidatum* Sieb. et Zucc.	5% DSS-induced C57BL/6 mice; 30 ng/mL TGF-β- induced colonocytes	↓ colon shortening, ↑ ZO-1 and Occludin; targeted MDM2/P53/PUMA axis to inhibit the apoptosis	In vivo and In vitro	[84]
			5% DSS-induced BALB/c mice; colon tissue culture in vitro	↓ weight loss, ↓ colon shortening, ↓ DAI; ↓ TNF-α, ↓ IL-6, ↓ IL-1β, ↓ IL-8, ↑ IL-10; improved the intestinal flora structure; suppressed PI3K/AKT/VEGFA	In vivo and In vitro	[41]
Terpenoids	Celastrol	*Tripterygium wilfordii* Hook. f., *Celastrus orbiculatus* Thunb.	3% DSS-induced BALB/c mice; naive CD4^+^ T cell differentiation model	↓ weight loss, ↓ colon shortening, ↓ DAI; ↓ TNF-α, ↓ IL-6, ↓ IL-1β, ↓ IL-17, ↓ IFN-γ, ↑ TGF-β, ↑ IL-10; rectified the Treg/Th1 and Treg/Th17 balances, modulated microbiota structure and metabolome	In vivo and In vitro	[85]
	Anemoside B4	*Pulsatilla chinensis* (Bge.) Regel	3% DSS-induced C57BL/6 mice; 0.1% DSS-induced intestinal organoids	↓ weight loss, ↓ colon shortening, ↓ DAI; ↓ TNF-α, ↓ IL-6, ↓ IL-1β, ↓ IL-17; ↑ ZO-1, MUC2 and Occludin; regulated the gut microbiome, increased butyric acid production, and activated AhR	In vivo and In vitro	[86]
			3% DSS-induced C57BL/6J mice; TNBS solution (80 mg/kg in 40% ethanol *v*/*v*)-instilled SD rats; 1 μg/mL LPS- stimulated THP-1 and Caco-2 cells	↓ weight loss, ↓ colon shortening, ↓ DAI; ↓ TNF-α, ↓ IL-6, ↓ IL-1β; inhibited PC enzyme activity, regulated pyruvate metabolism, and inhibited NF-κB	In vivo and In vitro	[87]
	18β-Glycyrrhetinic Acid	*Glycyrrhiza uralensis* Fisch.	250 μM TNBS and 10 μg/mL LPS-induced Caco-2 cells	↓ TNF-α; ↑ ZO-1 and Occludin; activated Wnt/β-catenin	In vitro	[88]
	Pristimerin	*Tripterygium wilfordii* Hook. f., *Celastrus orbiculatus* Thunb.	2.5% DSS-induced C57BL/6 mice; 3% DSS-induced Caco-2 cells; 0.5 μg/mL LPS-stimulated RAW 264.7 cells	↓ weight loss, ↓ colon shortening, ↓ DAI; ↓ TNF-α, ↑ IL-10, ↑ IL-22, ↑ SOD, ↓ MPO, ↓ MDA; ↑ Claudin-1 and Occludin; reshaped the gut microbiota and regulated host metabolism	In vivo and In vitro	[89]
	Ursolic acid	*Cornus officinalis* Sieb. et Zucc., *Ligustrum lucidum* Ait.	3% DSS-induced C57BL/6J mice	↓ weight loss, ↓ colon shortening, ↓ DAI; ↓ IL-1β, ↓ TNF-α, ↓ IL-6, ↓ IL-17A, ↑ TGF-β, ↑ IL-10; ↑ ZO-1 and Occludin. restored the Treg/Th17 balance; inhibited *E. coli* and reduced LPS levels through l-histidine	In vivo	[90]
	Asiatic acid	*Centella asiatica* (L.) Urb.	2.5% DSS-induced C57BL/6 mice; 1 μg/mL LPS-stimulated RAW 264.7 cells	↓ weight loss, ↓ colon shortening, ↓ DAI; ↓ IL-1β, ↓ TNF-α, ↓ IL-6, ↑ SIgA, ↑ IL-22, ↓ IL-23; ↑ Claudin-1 and MUC2; reshaped the gut microbiota, regulated γ-Glu-Tyr and cAMP/PKA/NF-κB	In vivo and In vitro	[91]
	Akebia saponin D	*Dipsacus asper* Wall. ex Henry	2.5% DSS-induced C57BL/6 mice; 1 μg/mL LPS-stimulated NCM460 and HT29 cells	↓ weight loss, ↓ colon shortening, ↓ DAI; ↓ IL-1β, ↓ TNF-α, ↓ IL-6, ↑ IL-10, ↑ ZO-1, Claudin-1 and Occludin; modulated gut *Akkermansia muciniphila* and promoted indole-3-carbinol; enhanced the proportions of Hmgb2^+^ TACs and Muc2^+^ GCs; suppressed MEK/ERK/AP-1	In vivo and In vitro	[92]
	Isodeoxyelephantopin	*Elephantopus scaber* L.	3% DSS-induced C57BL/6 mice; 100 ng/mL LPS and 2.5 mM ATP-stimulated THP-1 cells	↓ weight loss, ↓ colon shortening, ↓ DAI; ↑ MUC2 and Occludin; ↓ IL-1β, ↓ TNF-α, ↓ IL-6, ↓ IL-18; attenuated the TXNIP-NLRP3 interaction, inhibited NLRP3, reduced the cleavage of pro-caspase-1 and pro-IL-1β	In vivo and In vitro	[93]
	Ganoderic acid A	*Ganoderma lucidum* (Leyss. ex Fr.) Karst.	3% DSS-induced C57BL/6 mice; 1 μg/mL LPS-stimulated RAW264.7 cells; NCM460 cells treated with condi-tioned medium from RAW264.7 cells	↓ weight loss, ↓ colon shortening, ↓ DAI; ↓ IL-1β, ↓ TNF-α, ↓ IL-6; ↑ ZO-1, α-catenin and Occludin; decreased RORA, inhibited Th17 cells from secreting IL-17 and reduced M1 macrophage polarization	In vivo and In vitro	[43]
			2.5% DSS-induced C57BL/6 mice	↓ weight loss, ↓ colon shortening, ↓ DAI; ↑ ZO-1, Claudin-1, MUC2 and Occludin; ↓ iNOS, ↓ IL-1β, ↓ COX-2, ↓ IL-6, ↓ TNF-α; modulated the gut microbiota composition, increased 3-IAld, activated AhR and IL-22	In vivo	[44]
	Astragaloside IV	*Astragalus membranaceus* (Fisch.) Bge.	2.5% DSS-induced C57BL/6 mice; 100 µg/mL LPS-induced Caco-2 cells	↓ weight loss, ↓ colon shortening, ↓ DAI; ↑ ZO-1 and Occludin; ↓ IL-1β, ↓ IL-6, ↓ TNF-α; modulated the gut microbiota composition, inhibited PI3K/AKT	In vivo and In vitro	[94]
Polysaccharides	Astragalus polysaccharide	*Astragalus membranaceus* (Fisch.) Bge.	3% DSS-induced BALB/c mice	↓ weight loss, ↓ colon shortening, ↓ DAI; ↓ TNF-α, ↓ IL-6, ↓ IL-1β; ↓ MDA, ↑ T-AOC, ↑ GSH, ↑ SOD; ↑ ZO-1, Claudin-1 and Occludin; ↓ CD4^+^IL-17A^+^Th17 cells, ↑ CD25^+^Foxp3^+^Treg cells; restored SCFAs production, inhibited NF-κB	In vivo	[45]
			3% DSS-induced BALB/c mice	↓ weight loss, ↓ colon shortening, ↓ DAI; ↓ IL-2, ↓ IL-17A, ↓ IFN-γ, ↑ IL-4, ↑ IL-10, ↑ TGF-β1; regulated Treg cells and inhibited mTOR/HIF-1α	In vivo	[95]
			3% DSS-induced C57BL/6J mice; 1 μg/mL LPS-stimulated Caco-2 cells	↓ weight loss, ↓ colon shortening, ↓ DAI; ↓ TNF-α, ↓ IL-6, ↓ IL-1β, ↓ IL-2, ↓ IL-8, ↓ IL-18; ↑ IL-22, ↑ IL-4, ↑ IL-10; ↑ Claudin-1 and Occludin; activated AhR	In vivo and In vitro	[96]
			3% DSS-induced BALB/c mice	↓ colon shortening, ↓ colonic weight, ↓ colonic weight index, ↓ colonic weight/colonic length; restored mTh17/mTreg balance, regulated TIGIT/CD155	In vivo	[97]
	*Lycium barbarum* polysaccharide	*Lycium barbarum* L.	2% DSS-induced C57BL/6 mice; 1 μg/mL LPS-stimulated Caco-2 cells	↓ weight loss, ↓ colon shortening, ↓ DAI; ↓ MPO, ↓ TNF-α, ↓ IL-6, ↓ IL-1β, ↓ iNOS, ↑ IL-10; ↑ ZO-1, Claudin-2 and Occludin; modulated the abundance of probiotics and conditional pathogens, increased SCFAs production	In vivo and In vitro	[98]
			RAW264.7 cells (100 ng/mL LPS and 10 ng/mL IFN-γ stimulated M1 macrophages; 10 ng/mL IL-4 and 10 ng/mL IL-13 stimulated M2 macrophages); 2.5% DSS-induced C57BL/6 mice	↓ weight loss, ↓ colon shortening, ↓ DAI; ↓ NOS2, ↑ Arg-1; reduced p-STAT1 and promoted p-STAT6, regulated M1/M2 macrophage polarization	In vivo and In vitro	[47]
	Dendrobium polysaccharides	*Dendrobium nobile* Lindl.	2% DSS-induced C57BL/6J mice; 100 ng/mL LPS and 20 ng/mL IFN-γ induced M1 BMDMs, 20 ng/mL IL-4 induced M2 BMDMs	↓ IL-1β, ↓ TNF-α, ↓ IL-6, ↓ Nos2, ↑ CD206, ↑ Arg-1, ↑ Chil3; activated SENP1-SIRT3, promoted macrophage polarization	In vivo and In vitro	[46]
			3% DSS-induced C57BL/6J mice; 21.6 mg/mL DSS-induced NCM460 cells	↓ weight loss, ↓ colon shortening, ↓ DAI; ↓ TNF-α, ↓ IL-6, ↓ IL-1β, ↓ IL-17, ↓ IL-23; modulated *Lachnoclostridium edouardi*, increased SCFAs, and activated PPARγ to inhibit NF-κB	In vivo and In vitro	[99]
			2% DSS-induced C57BL/6 mice	↓ weight loss, ↓ colon shortening, ↓ DAI; ↓ TNF-α, ↓ IL-6, ↑ IL-10; ↑ ZO-1 and Occludin; modulated the gut microbiota, regulated purine and nicotinate/nicotinamide metabolism	In vivo	[100]
	Abelmoschi Corolla Polysaccharides	*Abelmoschus manihot* (L.) Medicus	2.5% DSS-induced C57BL/6 mice	↓ weight loss, ↓ colon shortening, ↓ DAI; ↓ TNF-α, ↓ IL-6, ↓ IL-1β; ↑ ZO-1 and Occludin; modulated the gut microbiota, increased 7-ketodeoxycholic acid, regulated FXR/STAT3	In vivo	[101]
	Ginseng polysaccharides	*Panax ginseng* C. A. Mey.	2.5% DSS-induced C57BL/6 mice	↓ weight loss, ↓ colon shortening, ↓ DAI; ↓ MPO, ↓ TNF-α, ↓ IL-6, ↓ IL-1β, ↓ IFN-γ, ↓ MCP-1, ↓ GM-CSF, ↑ IL-10; ↑ ZO-1, Claudin-1, and Occludin; modulated gut microbiota and mediated tryptophan metabolites, regulated HTR3A	In vivo	[102]

↑ indicates upregulation or promotion, and ↓ indicates downregulation or inhibition.

## Data Availability

No new data were created or analyzed in this study. Data sharing is not applicable.

## Abbreviations

The following abbreviations are used in this manuscript:

ABPP	Activity-based Protein Profiling
AhR	Aryl hydrocarbon receptor
BLI	Bio-layer interferometry
CC	Click chemistry
CETSA	Cellular Thermal Shift Assay
CO	Carbon monoxide
DARTS	Drug Affinity Responsive Target Stability
EVs	Extracellular vesicles
FMT	Fecal microbiota transplantation
IL	Interleukin
NDDS	Nano drug delivery system
NF-κB	Nuclear factor kappa B
NLRP3	NLR family pyrin domain containing 3
NO	Nitric oxide
PAL	Photoaffinity labeling
PI3K	Phosphatidylinositol 3-Kinase
ROS	Reactive oxygen species
SCFAs	Short-chain fatty acids
SILAC	Stable isotope labeling by amino acids
SPR	Surface Plasmon Resonance
TCM	Traditional Chinese medicine
Th17	T helper 17 cell
Treg	Regulatory T cell
UC	Ulcerative colitis
